# Expression, Purification, and Characterization of Interleukin-11 Orthologues

**DOI:** 10.3390/molecules21121632

**Published:** 2016-11-29

**Authors:** Andrei S. Sokolov, Alexei S. Kazakov, Valery V. Solovyev, Ramis G. Ismailov, Vladimir N. Uversky, Yulia S. Lapteva, Roman V. Mikhailov, Ekaterina V. Pavlova, Iana O. Terletskaya, Ludmila V. Ermolina, Sergei E. Permyakov, Eugene A. Permyakov

**Affiliations:** 1 Institute for Biological Instrumentation of the Russian Academy of Sciences, Institutskaya str., 7, Pushchino 142290, Moscow Region, Russia; 212sok@gmail.com (A.S.S.); fenixfly@yandex.ru (A.S.K.); ismailov_ramis@mail.ru (R.G.I.); lapteva.julia@gmail.com (Y.S.L.); permyakov.s@gmail.com (S.E.P.); 2 Biocad, Lubuchany 142380, Moscow Region, Russia; cvv@rambler.ru; 3 Department of Molecular Medicine and USF Health Byrd Alzheimer’s Research Institute, Morsani College of Medicine, University of South Florida, Tampa, FL 33612, USA; 4 Antherix, Institutskaya str., 7, Pushchino 142290, Moscow Region, Russia; romanuel@rambler.ru (R.V.M.); catarios-ksu@mail.ru (E.V.P.); ms.yterletskaya@mail.ru (I.O.T.); 5 JSC R-Pharm, Berzarina str., 19/1, Moscow 123154, Russia; lv.ermolina@rpharm.ru

**Keywords:** cytokines, interleukin-11, ubiquitin, cloning, bacterial expression, protein-protein interaction, ligand-receptor interaction, STAT3 signaling

## Abstract

Interleukin-11 (IL-11) is a multifunctional cytokine implicated in several normal and pathological processes. The decoding of IL-11 function and development of IL-11-targeted drugs dictate the use of laboratory animals and need of the better understanding of species specificity of IL-11 signaling. Here, we present a method for the recombinant interleukin-11 (rIL-11) production from the important model animals, mouse and macaque. The purified mouse and macaque rIL-11 interact with extracellular domain of human IL-11 receptor subunit α and activate STAT3 signaling in HEK293 cells co-expressing human IL-11 receptors with efficacies resembling those of human rIL-11. Hence, the evolutionary divergence does not impair IL-11 signaling. Furthermore, compared to human rIL-11 its macaque orthologue is 8-fold more effective STAT3 activator, which favors its use for treatment of thrombocytopenia as a potent substitute for human rIL-11. Compared to IL-6, IL-11 signaling exhibits lower species specificity, likely due to less conserved intrinsic disorder propensity within IL-6 orthologues. The developed express method for preparation of functionally active macaque/mouse rIL-11 samples is suited for exploration of the molecular mechanisms underlying IL-11 action and for development of the drug candidates for therapy of oncologic/hematologic/inflammatory diseases related to IL-11 signaling.

## 1. Introduction

**Figure 1:**
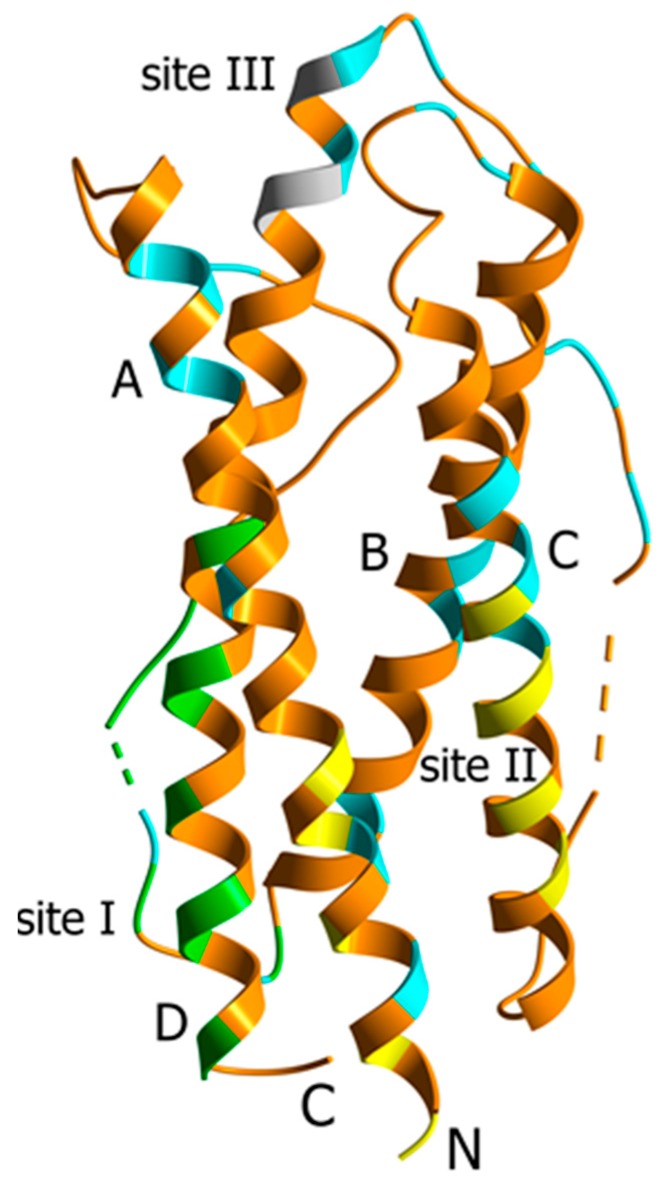
Crystal structure of human IL-11 (PDB entry 4MHL [20]). α-Helices A to D are labelled. Dashed lines mark the non-resolved regions. The residues comprising the proposed sites for IL-11 interaction with IL-11Rα (site I, green) and gp130 (site II, yellow; site III, grey) receptors [20] are indicated. The residues not conserved in either macaque or mouse IL-11 are shown in cyan (see Figure S1).

Interleukin-11 (IL-11; Adipogenesis inhibitory factor) is a hematopoietic cytokine belonging to the multifunctional interleukin-6 (IL-6) family of cytokines, which affect intracellular signaling pathways via interaction with the ubiquitously expressed signal-transducing receptor gp130 (except for IL-31) and a non-signaling cytokine-specific receptor [1,2,3,4]. IL-6 and IL-11 are the only IL-6 family members engaging two gp130 molecules for signaling. Having a sequence identity of 21%, IL-6 and IL-11 cytokines possess similar tertiary structures: a four-helix bundle motif with an up-up-down-down arrangement of α-helices (Figure 1). They exhibit similar, but not identical expression profiles (co-expressed in hepatocytes, B cells, macrophages and osteoclasts), both activate Jak/STAT and MAPK signaling pathways, exerting in some cases drastically different physiological effects, and both favor oncogenesis in numerous overlapping cancer types [1,2,5]. Targeted inhibition of IL-6 signaling is clinically used for treatment of rheumatoid arthritis and other autoinflammatory diseases (Tocilizumab), and has shown promising results in preclinical studies and clinical trials in various cancers [6,7,8,9]. Targeting of IL-11 signaling is by far less elaborated therapeutic approach. IL-11 is shown to be a key IL-6 family cytokine during gastrointestinal cancers [10], which is involved into progression of breast cancer [11], osteosarcoma [12], and many other cancers [1]. Several antibodies specific to either IL-11 or its cognate receptors are reported with efficacy in mouse models of several cancers [1,11]. Other ways for inhibition of IL-11 signaling are the use of IL-11 derivatives or mimetics competitively blocking IL-11 action (IL-11 Mutein, etc.), and application of decoy receptors (sIL-11Rα, sgp130-Fc as an inhibitor of IL-11 trans-signaling) [1,11,13,14,15,16]. The pharmacologic blocking of IL-11 signaling in cancer contradicts to clinical application of recombinant human IL-11 (Oprelvekin) for stimulation of bone marrow to prevent chemotherapy-induced thrombocytopenia. Meantime, the hematopoietic activity of IL-11 favors its use under other pathologic conditions requiring stimulation of hemopoiesis. Other medically useful IL-11 activities, such as growth factor activity (neuro- and osteoclastogenesis, inhibition of adipogenesis, cytoprotection, etc.), immunological activity (differentiation of immune cells, cytokine production by CD4^+^ T cells, anti-inflammatory activity, etc.) and female fertility control (reviewed in ref. [4,17,18]), are still waiting for their translation into clinical practice. For example, IL-11 is under investigation for treatment of rheumatoid arthritis, Crohn’s disease, and other inflammatory conditions [4,9,19].

The undergoing pre-clinical development of IL-11 antagonists, search for novel clinical applications of IL-11 and its derivatives, as well as further exploration of multifactorial physiological activities of IL-11 require the use of laboratory animals and respective knowledge of cross-reactivity between the human and animal protein orthologues involved in IL-11 signaling cascade. Importantly, IL-6 signaling is species-specific, since human IL-6 binds to murine IL-6 receptors, but not vice versa [2,21]. Meanwhile, the species specificity of IL-11 signaling is poorly studied. Human IL-11 efficiently interacts with 7TD1 cells co-expressing murine IL-11 receptors [22], but binding of animal IL-11 proteins to human IL-11 receptors is not reported. To promote studies of IL-11 signaling in animal models, here we present an express method for laboratory-scale bacterial expression and purification of functionally active IL-11 proteins from the conventional laboratory animal species, mouse (*Mus musculus*) and cynomolgus monkey (*Macaca fascicularis*) (sequence identity to human IL-11 of 87.6% and 93.8%, respectively—see Figure S1). The initial characterization of the cross-species reactivity shows that compared to IL-6 signaling IL-11 exhibits lower species specificity. Furthermore, the higher potency of macaque IL-11 to STAT3 activation in the genetically modified HEK293 cells could be therapeutically significant in treatment of thrombocytopenia.

## 2. Results and Discussion

Mammalian IL-11 typically lack Cys residues and posttranslational modifications, which favors use of bacterial expression systems for their production (although eukaryotic expression systems are proposed for human IL-11 [23,24]).

**Figure 2:**
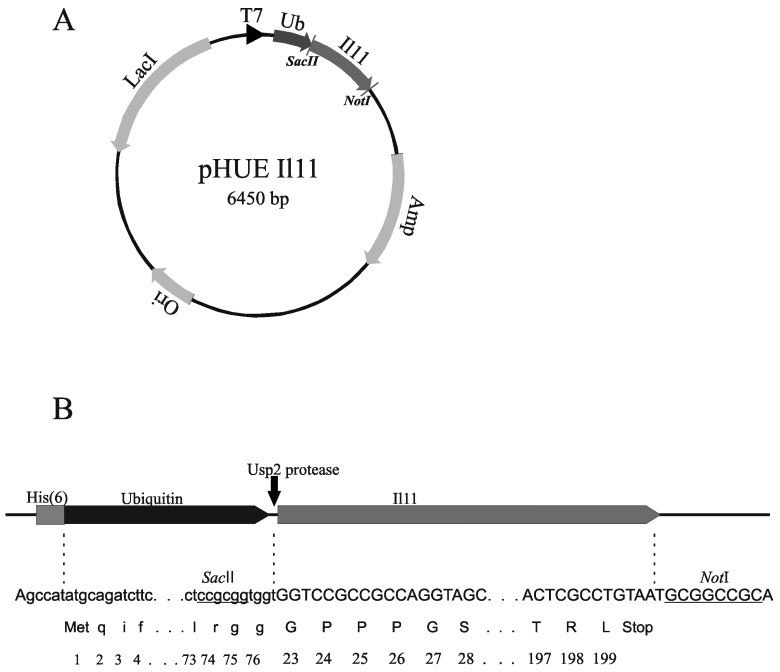
(**A**) Plasmid map of pHUE IL-11 vector showing the Ub coding region (black arrow), the IL-11 coding region (grey arrow), the T7 RNA polymerase promoter (black triangle) and other regions (shaded arrows), including ColE1 origin of replication (Ori), LacI repressor (LacI) and β-lactamase (Amp) genes. Arrows indicate the direction of transcription. The SacII/NotI restriction sites used for cloning of IL-11 gene are shown; (**B**) The schematic presentation of the Ub-IL-11 fusion protein. The black arrow indicates the site of hydrolysis by Usp2 protease. The nucleotide sequences of the 5′ end and the restrictions sites (macaque IL-11) are shown. The translation into the protein sequence is shown below. The numbering of protein residues corresponds to the non-processed protein (Swiss-Prot entry P20808).

Indeed, *Escherichia coli* (*E. coli*) cells are widely used for production of IL-11 as a mature protein lacking a signal peptide (human IL-11 [25,26]) or as a fusion protein (human [27,28,29,30] or murine IL-11 [31]). The predominance of the latter approach seems to be due to higher expression levels provided by the use of fusion constructs [27]. Following this trend, we expressed macaque and murine orthologues of IL-11 as a fusion with ubiquitin (‘Ub’), instead of thioredoxin, tumor necrosis factor-α or glutathione S-transferase, used by others [27,28,29,30,31]. This approach was previously validated for preparation of human IL-11 [32]. We cloned the nucleotide sequence encoding the mature murine or macaque IL-11, which similarly to Oprelvekin lacks Pro1 residue (Table S1), into the pHUE expression vector based on pET15b [33] between the *Sac*II and *Not*I restriction sites (Figure 2).

The IL-11 fusion with 6 × His-tagged ubiquitin was expressed in *E. coli* BL21(DE3)/pLacIRARE strain optimized for expression of the genes with rare *E. coli* codons (the case of IL-11—see Table S1). The soluble fusion protein was retrieved from the cells using French press, captured by Profinity IMAC resin (Bio-Rad Laboratories, Inc., Hercules, CA, USA) and eluted by imidazole solution. IL-11 was cleaved from the chimeric protein by Usp2 Ub-specific protease, cleaned from the His-tagged Ub and Usp2 protease by IMAC, and purified to homogeneity by gel filtration using Sephacryl S-100 HR (GE Healthcare, Chicago, IL, USA) medium.

**Figure 3:**
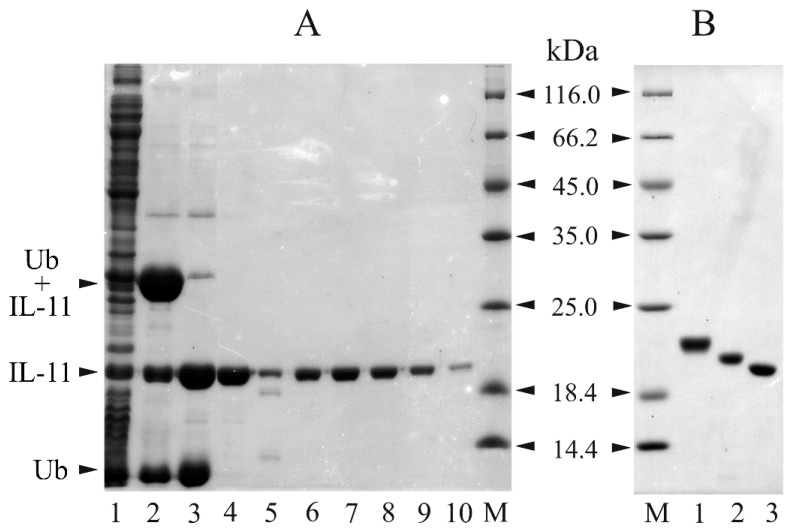
(**A**) Expression in *E. coli* BL21(DE3)/pLacIRARE strain and purification of murine IL-11 (the data for macaque IL-11 are similar), controlled by reducing SDS-PAGE (15%; staining by Coomassie brilliant blue R-250). Lane 1, soluble extract of the cells expressing the Ub-IL-11 fusion; lanes 2 and 3, the affinity-purified Ub-IL-11 fusion sample before and after the cleavage by Usp2 protease, respectively; lane 4, the IL-11 sample after cleaning from ubiquitin and Usp2 protease; lanes 5–10, the protein fractions after the gel filtration using Sephacryl S-100 HR medium (the sample shown in lane 5 was discarded). ‘M’ denotes the lanes with molecular mass standards (the masses in kDa are indicated in-between the panels **A** and **B**); (**B**) SDS-PAGE of the purified recombinant human, macaque and murine IL-11 (lanes 1, 2 and 3, respectively).

Figure 3 shows progress of the fusion expression, IL-11 cleavage from the fusion protein and subsequent purification stages, controlled by SDS-PAGE. The soluble cellular extract and the affinity-purified chimeric sample reveal spontaneous degradation of the Ub-IL-11 fusion giving rise to the fragments with molecular masses resembling those for Ub and IL-11 (Figure 3A, lanes 1 and 2).

This phenomenon was reported for the ubiquitin fusion system [34] and seems to be due to intrinsic proteolytic activity of the Ub fusion (which explains presence of IL-11 in the lane 2), but could be due to presence in *E. coli* of Usp2-like deubiquitinating enzyme(s) [35]. Nevertheless, the resulting purified samples of recombinant IL-11 (‘rIL-11’) exhibit a single major (>95%) band with molecular mass of 20 kDa (Figure 3B; predicted mass of 19.0–19.3 kDa). Although the rIL-11 samples studied are very close to each other in the content and location of charged amino acid residues (differ by presence in the macaque protein of an additional Glu in position 30—see Figure S1), they demonstrate notably different electrophoretic mobility (Figure 3B). The yield of macaque and mouse rIL-11 was of 3 mg and 5 mg of protein per liter of cell culture, respectively.

**Figure 4:**
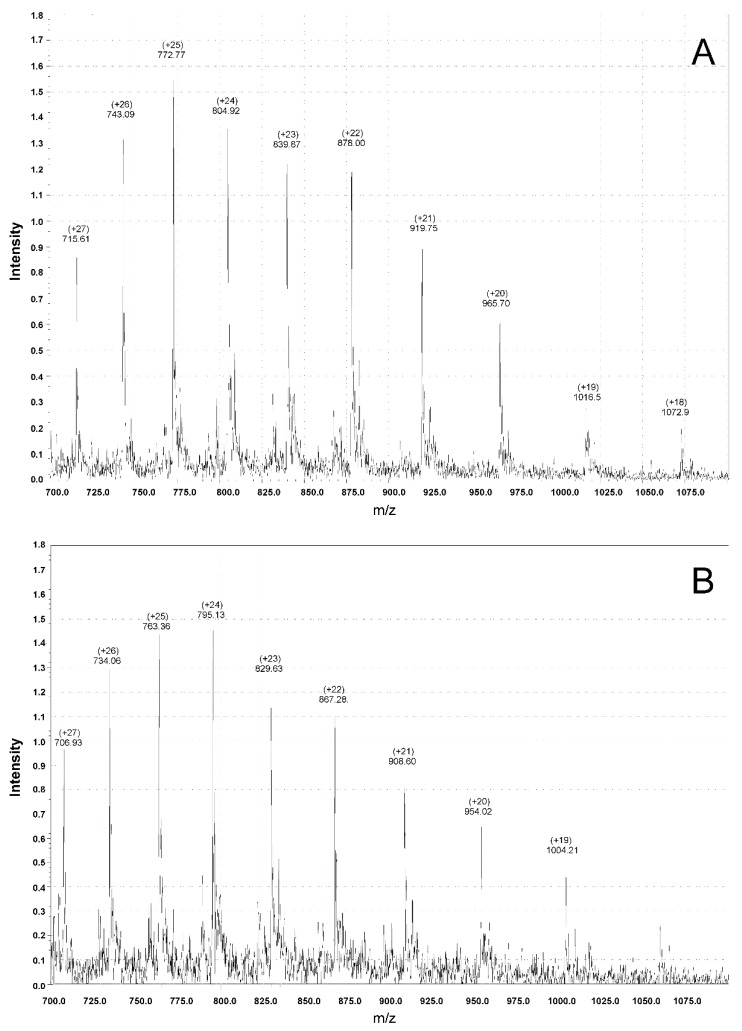
ESI-MS spectra of macaque (**A**) and murine (**B**) rIL-11 samples. *m*/*z* values and their respective relative charge values (+z) are indicated above the major peaks.

To confirm the precise excision of the His-tagged Ub from the Ub-IL-11 chimera, the resulting rIL-11 samples were studied by electrospray ionization mass spectrometry (ESI-MS). The ESI-MS spectra shown in Figure 4 reveal that both rIL-11 samples exhibit a single set of major peaks, corresponding to molecular mass values of macaque and murine rIL-11 of 19,292 Da (49%) and 19,057 Da (48%), respectively. The experimental estimates are in accord with the values expected for the rIL-11 not affected by post-translational modifications: 19,291.6 Da and 19,057.3 Da for macaque and murine proteins, respectively. Meanwhile, three minor protein components were detected for each of the samples (Table S3). Two of them for murine rIL-11 likely correspond to the sodium-bound protein forms, suggesting that 87% of the sample agree with the expected mass. Similarly, 70% of macaque rIL-11 possess the expected mass (Table S3). Overall, Usp2 protease ensures correct cleavage of the Ub-IL-11 fusion.

**Figure 5:**
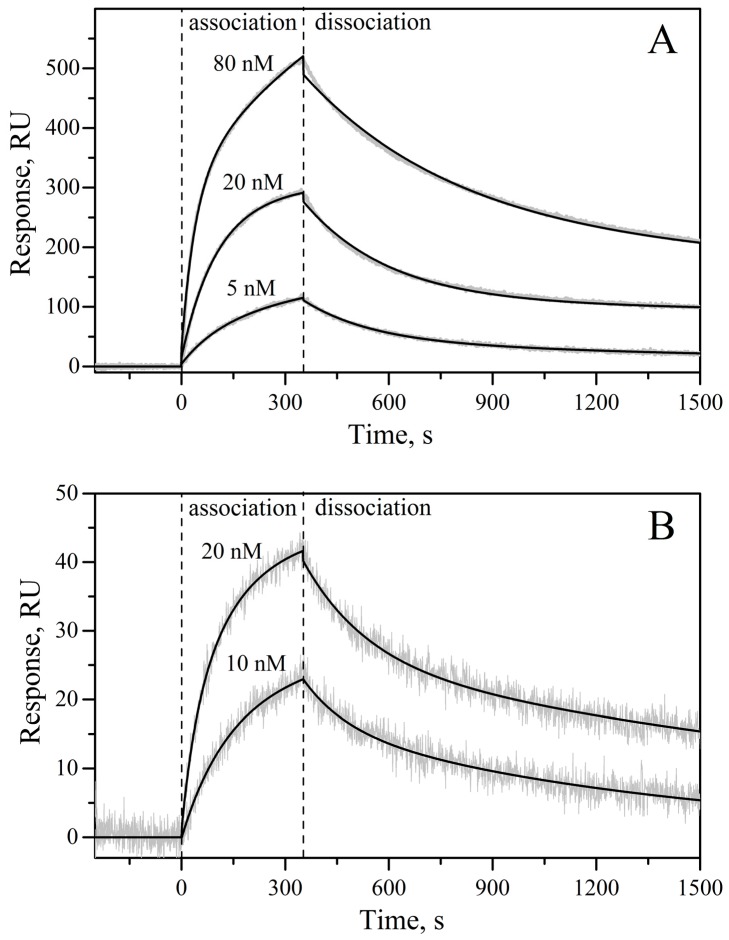
Kinetics of the interaction of macaque/murine rIL-11 with sIL-11Rα (panels **A** and **B**, respectively) at 25 °C, monitored by SPR spectroscopy (PBS, 0.05% TWEEN 20, pH 7.4 buffer). The biotinylated rIL-11 was immobilized on the surface of NLC sensor chip. Concentrations of sIL-11Rα used as an analyte are indicated above the curves. Grey curves are experimental, while black curves are theoretical, calculated according to the heterogeneous ligand model (1) (see Table 1 for the fitting parameters).

Functional activity of IL-11 includes its ability to recognize the cognate receptors and to induce the intracellular signaling mediated by gp130 receptor. To estimate rIL-11 affinity to interleukin-11 receptor subunit α (‘IL-11Rα’), the interaction between rIL-11 and recombinant extracellular domain of human IL-11Rα with a fused His-tag at the C-terminus (‘sIL-11Rα’) was studied by surface plasmon resonance (SPR) spectroscopy. The SPR sensograms for biotinylated rIL-11 immobilized on the surface of NLC sensor chip exhibit a concentration-dependent association-dissociation pattern in response to 5–80 nM sIL-11Rα (Figure 5). The kinetic SPR data are well approximated by the *heterogeneous ligand* model (1) with equilibrium dissociation constants, *K_d_*, of 4.2 nM and 8.9 nM for macaque rIL-11 and 2.2 nM and 14.6 nM for murine rIL-11 (Figure 5, Table 1). The 1.6-fold higher affinity of macaque rIL-11 (*K_d_*_2_) is in line with its 1.6-fold lower kinetic dissociation constant, *k_d_*_2_. These estimates are close to the *K_d_* (7 nM and 21.9 nM) and *k_d_* (3.5 × 10^−4^ s^−1^ and 3.84 × 10^−3^ s^−1^) values previously measured by SPR for interaction of human rIL-11, immobilized on the chip surface by amine coupling, with sIL-11Rα [32].

**Figure 6:**
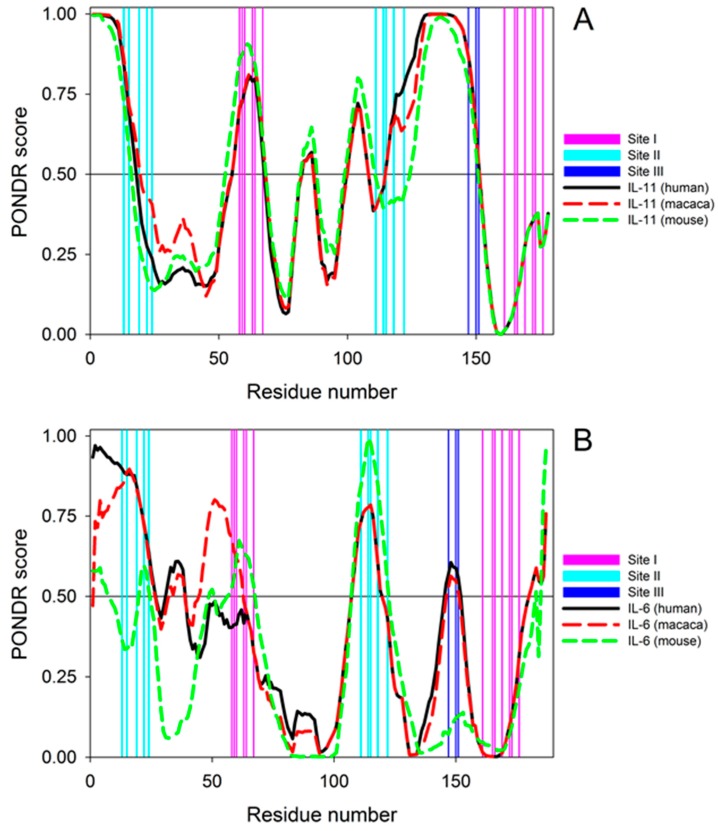
Predictions of per-residue disorder level for mature IL-11 (panel **A**) and IL-6 (panel **B**) orthologues from human (black solid lines), macaque (red dashed lines), and mouse (green dashed lines), using PONDR^®^ VLXT algorithm (http://pondr.com/). Positions of sites responsible for recognition of IL-receptors are shown as differently colored vertical bars (pink, cyan, and blue for sites I, II, and III, respectively).

Furthermore, similar *K_d_* value (10–50 nM) was reported for interaction between human IL-11 and FLAG-tagged extracellular domain of murine IL-11Rα immobilized on anti-FLAG labeled beads [16]. Thus, interaction of IL-11 with IL-11Rα receptor is less species-specific, compared to interaction of IL-6 with its receptors, since human IL-6 binds to mouse IL-6 receptors, but not vice versa [2,21]. The lack of mouse IL-6 binding to human IL-6 receptors can be ascribed to the drastic difference between mouse IL-6 and macaque/human IL-6 in disorder propensities of the critical protein regions (sites II and III), which is absent in the case of IL-11 orthologues (Figure 6). The same *K_d_* value (10 nM) was reported for affinity of murine IL-11 to mouse Ba/F3 cells expressing murine IL-11Rα, but not gp130 receptor [36]. Hence, full-sized IL-11Rα receptor and its extracellular domain possess nearly equal affinity to IL-11.

**Figure 7:**
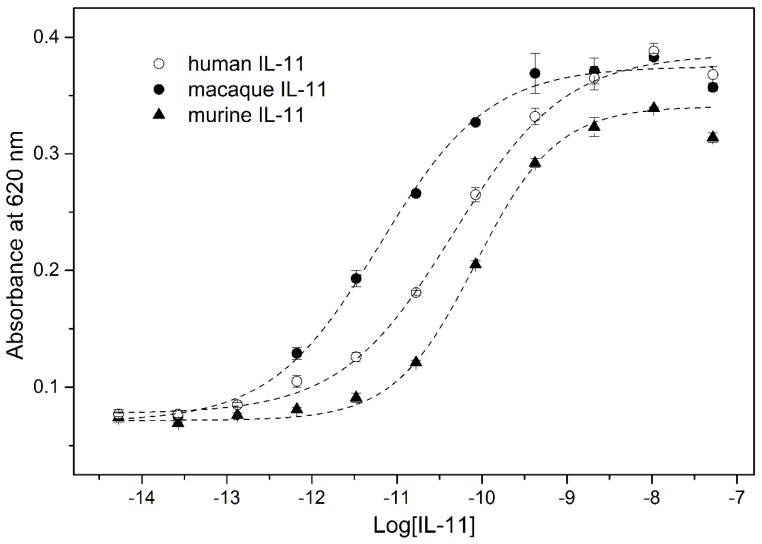
IL-11-induced STAT3 activation in a HEK-Blue™ IL-6 cell line transfected with human IL-11Rα gene, monitored by STAT3-induced secretion of SEAP (QUANTI-Blue™ assay) for murine, macaque and human rIL-11. Standard deviations estimated from the duplicate measurements are shown. The dashed curves are theoretical fits to the experimental curves using Boltzmann function.

Since the macaque/mouse rIL-11 proteins exhibit the affinities to the extracellular domain of IL-11Rα receptor (Table 1) equivalent to that reported in literature, they are expected to have analogous affinity to full-sized IL-11Rα receptor. If this interaction is accompanied by efficient engagement of gp130 receptor, the rIL-11 samples should initiate intracellular signaling. To verify this suggestion, an ability of the rIL-11 to activate Signal transducer and activator of transcription 3 (STAT3) was studied using modified HEK-Blue™ IL-6 cells line, stably transfected with human IL-11Rα gene as described in ref. [32]. The secreted embryonic alkaline phosphatase (SEAP) is liberated in response to STAT3 activation and detected using QUANTI-Blue™ colorimetric assay. An increase in rIL-11 concentration is accompanied by SEAP secretion, which follows STAT3 activation with EC_50_ values of 6 pM, 46 pM and 84 pM for macaque, human and murine rIL-11, respectively (Figure 7). The EC_50_ values for human and murine rIL-11 are in accord with the previous reports on IL-11-dependent proliferation of B9 and 7TD1 cells (EC_50_ of (20–40) pM [22,37]). The about an order of magnitude higher activity of macaque rIL-11 relative to human and murine rIL-11 could be both due to its higher affinity to gp130 receptor (see Table 1 for affinity to sIL-11Rα) and more efficient activation of IL-11 receptor(s). The proximity of EC_50_ value for macaque rIL-11 to plasma IL-11 level ((1.3–2.3) pM [38]) makes it a potent STAT3 activator. Since macaque rIL-11 has high sequence identity (93.8%) to its human counterpart (Oprelvekin) that was used in clinical practice for prevention of chemotherapy-induced thrombocytopenia, it represents a potentially more effective substitution for Oprelvekin.

Summing up, the macaque and mouse recombinant IL-11 samples demonstrate high potencies to human IL-11Rα recognition and STAT3 activation, comparable to or exceeding those for human rIL-11. The native-like functional activity of the rIL-11 seems to be due to mainly intact proposed sites of recognition of IL-11 receptors (sites I to III, see ref. [20]), except for A63T substitution in murine rIL-11, affecting the IL-11Rα-binding site (see Figure S1). High structural stability of human IL-11 molecule [39] could impede allosteric influence of the amino acid substitutions located beyond the sites I-III on recognition of IL-11 receptors and their activation.

In order to shed some lights on the observed differences in the cross-species reactivity between the IL-11 and IL-6 proteins, we investigated the per-residue intrinsic disorder propensities of these proteins from *Homo sapiens*, *Macaca fascicularis*, and *Mus musculus*. The need for this analysis is justified by the known facts that intrinsically disordered proteins (IDPs) and hybrid proteins containing both ordered and intrinsically disordered protein regions (IDPRs) [40] are very common in nature [41,42,43,44,45,46], and due to their lack of unique 3D-structures, these proteins/regions can carry out numerous crucial biological functions, such as signaling, regulation, and recognition [45,47,48,49,50,51,52,53,54,55,56,57,58,59,60,61,62,63,64,65,66].

Furthermore, disordered regions are commonly involved in protein-protein interactions [50,52,67,68,69,70,71]. Results of disorder propensity analysis are represented in Figure 6 that clearly shows the presence of noticeable difference in the conservation of disorder profiles of IL-11 and IL-6 proteins. In fact, irrespectively of their origin and in agreement with available structural information both IL-11 and IL-6 are predicted to be mostly ordered proteins that are expected to have IDPRs. Figure 6A illustrates that despite their relatively low sequence identity, IL-11 proteins from three organisms are characterized by very similar disorder profiles, indicating that the peculiarities of disorder distribution within their sequences are evolutionary conserved and, therefore, suggesting that intrinsic disorder-related features might be of functional importance.

In agreement with this hypothesis, Figure 6A shows that intrinsic disorder propensities of the sites I-III involved in interactions IL-11 receptors are rather similar in these three proteins. The different situation is observed for IL-6 proteins from *Homo sapiens*, *Macaca fascicularis*, and *Mus musculus* (see Figure 6B), which are characterized by the noticeable divergence of their disorder profiles and by the presence of very pronounced difference in the disorder propensities of all three sites involved in interaction with receptors. These results suggest that IL-11 proteins of different origin are expected to similarly interact with the receptors, whereas, due to the significant differences in disorder propensity of their binding sites, IL-6 proteins should possess noticeable differences in the cross-species reactivity.

## 3. Experimental Section

### 3.1. Materials

The deubiquitinating protease Usp2 was prepared as described in [33]. sIL-11Rα was from Sino Biological, Inc. (#10252-H08H, Beijing, China). pHUE and pHUsp2-cc vectors were kindly provided by Dr. Rohan T. Baker [33]. *E. coli* strain BL21(DE3) was purchased from New England Biolabs (Ipswich, MA, USA). *E. coli* strain Top10, *Sac*II and *Not*I restriction enzymes, T4 DNA ligase, Taq DNA polymerase and dNTPs were from Thermo Fisher Scientific Inc. (Waltham, MA, USA). Components of 2YT media, SDS and molecular mass markers for SDS-PAGE were from Helicon (Moscow, Russia). Coomassie Brilliant Blue R-250 was from Merck KGaA (Darmstadt, Germany). KH_2_PO_4_, Na_2_HPO_4_, NaOH, imidazole and HPLC grade acetonitrile were from Panreac Química S.L.U. (Barcelona, Spain). IPTG was from Serva Electrophoresis GmbH (Heidelberg, Germany). PMSF was purchased from Amresco^®^ LLC (Solon, OH, USA). Profinity IMAC resin and ProteOn™ NLC sensor chip were from Bio-Rad Laboratories, Inc. (Hercules, CA, USA). Sephacryl S-100 HR was purchased from GE Healthcare (Chicago, IL, USA). Formic acid and biotin 3-sulfo-N-hydroxysuccinimide ester sodium salt were from Sigma-Aldrich^®^ Co. (St. Louis, MO, USA). HEK-Blue™ IL-6 strain and QUANTI-Blue™ colorimetric enzyme assay were from InvivoGen (San Diego, CA, USA). All buffers and other solutions were prepared using either distilled or ultrapure water.

### 3.2. Design of Plasmids

The nucleotide sequence encoding mature IL-11 from *Macaca fascicularis* or *Mus musculus* (Swiss-Prot entries P20808 and P47873, respectively) without Pro1 residue (177 amino acid residues; Table S1) was cloned into His-tagged Ub Expression (pHUE) vector designed on the basis of pET15b for protein expression in *Escherichia coli* (*E. coli*) cells as a histidine-tagged ubiquitin fusion [33] (Figure 2). The pHUE vector contains the inducible T7 RNA polymerase promoter and β-lactamase gene for ampicillin resistance. The primers complementary to the ends of IL-11 open reading frame and containing *Sac*II and *Not*I restriction sites (Table S2) were used for PCR amplification of the IL-11 gene. The PCR fragments were digested with *Sac*II and *Not*I and cloned into the pHUE vector. The PCR and gene cloning were carried out using standard protocols. Nucleotide sequences of the resulting pHUE IL-11 vectors were verified by restriction analysis and automatic DNA sequencing of the inserts.

### 3.3. Preparation of Recombinant IL-11 Samples

Preparation of recombinant macaque/murine IL-11 samples was performed as described for human IL-11 [32]. The fusion between IL-11 and His-tagged ubiquitin (‘Ub’) was expressed in *E. coli* BL21(DE3)/pLacIRARE strain designed for expression of the genes with rare *E. coli* codons (the case of IL-11—refer to Table S1). Aside from the gene for T7 RNA polymerase under control of the lacUV5 promoter this strain contains pLacIRARE plasmid (Novagen, Darmstadt, Germany) with the gene for *lac* repressor and the tRNA genes for the rare *E. coli* codons encoding Arg, Ile, Gly, Leu and Pro, except for Arg CGA/CGG. *E. coli* BL21(DE3)/pLacIRARE cells were transformed with the pHUE IL-11 plasmid. The cells were grown at 37 °C in 1 L of 2YT medium with 100 µg/mL ampicillin, shaking at 200 rpm, until optical density at 600 nm reached 0.8–1.0 AU. IL-11 expression was induced at 25 °C by 0.2 mM IPTG. The cells were grown for 3 h, harvested by centrifugation at 5000 × *g* for 15 min at 4 °C, resuspended in 30 mL of lysis buffer (50 mM phosphate, 3 mM imidazole, 0.5 mM PMSF, 0.5 M NaCl, pH 8.0), and disintegrated using a French press. The lysate was centrifuged at 25,000 × *g* for 40 min at 4 °C, followed by incubation with 2 mL of Profinity IMAC resin for 4 h at 4 °C (shaking at 50 rpm). The resin was washed with 50 ml of buffer A (50 mM phosphate, 1 M NaCl, pH 8.0) and 50 ml of buffer A with 5 mM imidazole, packed into column and washed with buffer A. The protein was eluted with 10 mM phosphate, 150 mM NaCl, 300 mM imidazole, pH 7.5 buffer. The fractions containing Ub-IL-11 chimera were joined and treated with Usp2 Ub-specific protease (50-100-fold molar excess of the chimera over the enzyme, 16 h at 4 °C), followed by dialysis against phosphate buffered saline (PBS). The His-tagged Ub and Usp2 were removed from the sample via passage through the Profinity IMAC column. The IL-11 sample was further purified using HiPrep 26/60 Sephacryl S-100 HR gel filtration column equilibrated with 10 mM phosphate, 150 mM NaCl, pH 7.5 buffer (flow rate of 1 mL/min). The purified protein was dialyzed at 4 °C against PBS, aliquoted and stored at −20 °C. Purity of the recombinant IL-11 (‘rIL-11’) samples was controlled by reducing SDS-PAGE (15%) and staining by Coomassie brilliant blue R-250 [72]. rIL-11 concentration was determined spectrophotometrically, using molar extinction coefficient at 280 nm of 17,990 M^−1^cm^−1^, calculated from the amino acid content according to [73].

### 3.4. Mass Spectrometry Measurements

Protein molecular masses were determined by electrospray ionization mass spectrometry (ESI-MS). The sample of 0.1–0.5 µM rIL-11 in 40:60 (*v*/*v*) mixture of deionized water and acetonitrile (10 mM formic acid) was directly infused at rate of 40 µL/min into LCMS-2010EV (Shimadzu Co., Kyoto, Japan) mass detector (ESI probe coupled to a single quadrupole detector). Mass spectrometry spectra were collected in positive mode from 500 to 1500 *m*/*z* at scan speed 125 amu/s, detector voltage 1.5 kV, and nebulizing nitrogen flow 1.2 L/min. Instrument calibration was carried out using horse heart myoglobin (Sigma-Aldrich^®^ Co.).

### 3.5. Protein Biotinylation

rIL-11 biotinylation was carried out according to Thermo instructions to EZ-Link™ Sulfo-NHS-LC-Biotinylation Kit. Biotinylation degree was controlled using Thermo Pierce™ Biotin Quantitation Kit and Thermo HABA Calculator (http://www.piercenet.com/haba/habacalcmp.cfm). Biotinylation degree of the rIL-11 samples was about two biotin molecules per protein molecule.

### 3.6. Surface Plasmon Resonance Studies

Surface plasmon resonance (SPR) measurements were performed at 25 °C using Bio-Rad ProteOn™ XPR36 instrument (Hercules, CA, USA). Biotinylated rIL-11 was used as a ligand: 30 µg/mL rIL-11 in a running buffer (PBS, 0.05% TWEEN 20, pH 7.4) was immobilized on the surface of Bio-Rad ProteOn™ NLC sensor chip covered by NeutrAvidin (up to 2000 resonance units, RUs). sIL-11Rα was used as an analyte: 5–80 nM of the protein in the running buffer flowed over the chip at rate of 30 µL/min for 350 s, followed by flushing the chip with the running buffer for 3600 s. The double-referenced SPR sensograms were fitted according to a *heterogeneous ligand* model, which assumes existence of two populations of the ligand (‘L_1_‘ and ‘L_2_’) that bind single analyte molecule (‘A’):

(1) $$L_{1}+A\overset{K_{d1}}{\underset{k_{d1}}{↔}}L_{1}A;L_{2}+A\overset{K_{d2}}{\underset{k_{d2}}{↔}}L_{2}A$$

where *K_d_* and *k_d_* refer to equilibrium and kinetic dissociation constants, respectively. *K_d_*, *k_d_* and *R_max_* (maximum response) values were evaluated using Bio-Rad ProteOn Manager™ v.3.1 software. Standard deviations of the parameters were calculated from the estimates for several analyte concentrations. The sensor chip surface was regenerated by passage of 0.5% SDS water solution for 50 s.

### 3.7. IL-11-Induced STAT3 Activation Assay

rIL-11-induced activation of Signal transducer and activator of transcription 3 (STAT3) in Human Embryonic Kidney 293 (HEK293) cells was monitored using HEK-Blue™ IL-6 cells stably transfected with human IL-11Rα gene as described in [32]. The experimental data were described using Boltzmann function, as implemented in OriginPro 9.0 (OriginLab Corporation (Northampton, MA, USA) software.

### 3.8. Per-residue Protein Disorder Predictions

Amino acid sequences of IL-11 and IL-6 from *Homo sapiens*, *Macaca fascicularis*, and *Mus musculus* were retrieved from Swiss-Prot. These sequences without signal peptides are shown in Figure S2 (see Supplementary Materials). For IL-11 proteins from *Homo sapiens*, *Macaca fascicularis*, and *Mus musculus*, they correspond to the residues 22–199 of the Swiss-Prot entries P20809, P20808, and P47873, whereas for the IL-6 proteins from the same species, the analyzed sequences correspond to residues 26–212 of the Swiss-Prot entries P05231 and P79341, and residues 25–212 of the entry P08505, respectively. The intrinsic disorder propensities of the mature forms of these query proteins (IL-11 and IL-6 from *Homo sapiens*, *Macaca fascicularis*, and *Mus musculus*) were analyzed using PONDR^®^ VLXT, which is not the most accurate predictor of intrinsic disorder, but has a high sensitivity to local sequence peculiarities, which are often associated with the disorder-based interaction sites [52,74].

## 4. Conclusions

The described herein method for the laboratory-scale bacterial production of macaque and mouse rIL-11 is based on the use of ubiquitin fusion system validated for multiple eukaryotic proteins [33,34] and previously successfully used for preparation of human rIL-11 [32]. The application of His-tagged ubiquitin fusion ensures rapid and efficient protein purification in milligram quantities, the amounts easily covering research needs, since vital IL-11 concentrations lie in picomolar region [38]. The resulting rIL-11 samples are mainly homogeneous with respect to their N-terminus, as confirmed by mass spectrometry (Figure 4, Table S3). The proteins efficiently interact with the extracellular domain of human IL-11Rα, as evidenced by SPR spectroscopy (Figure 5, Table 1), and activate STAT3 signaling in the model HEK293 cells co-expressing human IL-11 receptors (Figure 7). This implies that species specificity of IL-11 signaling is lower compared to that for IL-6, which could be due to less conserved protein disorder propensity within IL-6 orthologues (Figure 6). Functional activities of the rIL-11 orthologues are close to or higher than those for human rIL-11, which enables their use for studies of multifaceted activity of IL-11 under normal and pathological physiological conditions, as well as for development of the drug candidates targeted against IL-11 or its receptors. The latter cover wide spectrum of oncologic, hematologic and inflammatory diseases intimately linked to IL-11 signaling [1,4,9,19]. The increased with regard to human rIL-11 ability of macaque rIL-11 to STAT3 activation and its high homology to the human protein indicate potential usefulness of macaque rIL-11 for treatment of thrombocytopenia and similar pathologic conditions requiring stimulation of hemopoiesis. Meanwhile, further studies are required to validate this suggestion.

## Figures and Tables

**Table 1:** Kinetic and equilibrium dissociation constants describing the SPR data (Figure 5) on interaction of macaque/mouse rIL-11 with sIL-11Rα according to the heterogeneous ligand model (1). Standard deviations estimated from the global fit of three kinetic SPR curves measured at different analyte concentrations are indicated.

rIL-11 Sample	*k_d_*_1_, s^−1^	*K_d_*_1_, nM	R_max1_	*k_d_*_2_, s^−1^	*K_d_*_2_, nM	R_max2_
macaque	(8.15 ± 0.03) × 10^−4^	4.2 ± 2.6	352	(3.8 ± 1.4) × 10^−3^	8.9 ± 1.8	203
mouse	(7.0 ± 2.5) × 10^−4^	2.2 ± 0.7	31	(6.1 ± 1.3) × 10^−3^	14.6 ± 0.8	19

## Supplementary Materials

Supplementary materials can be accessed at: http://www.mdpi.com/1420-3049/21/12/1632/s1.

## Abbreviations

*E. coli*, *Escherichia coli*; IL, interleukin; Ub, ubiquitin; rIL-11, recombinant interleukin-11 lacking Pro1 residue of the mature protein; IL-11Rα, interleukin-11 receptor subunit α; sIL-11Rα, recombinant extracellular domain of human interleukin-11 receptor subunit α (M1-V363) with a fused polyhistidine tag at the C-terminus; gp130, interleukin-6 receptor subunit β; STAT3, Signal transducer and activator of transcription 3; HEK293, Human Embryonic Kidney 293 cell line; SEAP, secreted embryonic alkaline phosphatase; IPTG, isopropyl β-D-1-thiogalactopyranoside; PMSF, phenylmethanesulfonyl fluoride; PBS, phosphate-buffered saline; SDS, sodium dodecyl sulfate; PCR, polymerase chain reaction; IMAC, immobilized metal affinity chromatography; PAGE, polyacrylamide gel electrophoresis; ESI-MS, electrospray ionization mass spectrometry; SPR, surface plasmon resonance; RU, resonance unit in surface plasmon resonance spectroscopy.

## References

[1] Putoczki T.L., Ernst M. (2015). IL-11 signaling as a therapeutic target for cancer. Immunotherapy.

[2] Garbers C., Scheller J. (2013). Interleukin-6 and interleukin-11: Same same but different. Biol. Chem..

[3] Paul S.R., Bennett F., Calvetti J.A., Kelleher K., Wood C.R., O’Hara R.M., Leary A.C., Sibley B., Clark S.C., Williams D.A. (1990). Molecular cloning of a cdna encoding interleukin 11, a stromal cell-derived lymphopoietic and hematopoietic cytokine. Proc. Natl. Acad. Sci. USA.

[4] Negahdaripour M., Nezafat N., Ghasemi Y. (2016). A panoramic review and in silico analysis of IL-11 structure and function. Cytokine Growth Factor Rev..

[5] Taniguchi K., Karin M. (2014). IL-6 and related cytokines as the critical lynchpins between inflammation and cancer. Semin. Immunol..

[6] Schaper F., Rose-John S. (2015). Interleukin-6: Biology, signaling and strategies of blockade. Cytokine Growth Factor Rev..

[7] Tanaka T., Narazaki M., Ogata A., Kishimoto T. (2014). A new era for the treatment of inflammatory autoimmune diseases by interleukin-6 blockade strategy. Semin. Immunol..

[8] Kumari N., Dwarakanath B.S., Das A., Bhatt A.N. (2016). Role of interleukin-6 in cancer progression and therapeutic resistance. Tumour Biol..

[9] Yuzhalin A.E., Kutikhin A.G. (2015). IL-6 family and cancer. Interleukins in Cancer Biology.

[10] Putoczki T.L., Thiem S., Loving A., Busutti R.A., Wilson N.J., Ziegler P.K., Nguyen P.M., Preaudet A., Farid R., Edwards K.M. (2013). Interleukin-11 is the dominant IL-6 family cytokine during gastrointestinal tumorigenesis and can be targeted therapeutically. Cancer Cell.

[11] Johnstone C.N., Chand A., Putoczki T.L., Ernst M. (2015). Emerging roles for IL-11 signaling in cancer development and progression: Focus on breast cancer. Cytokine Growth Factor Rev..

[12] Lewis V.O. (2014). IL-11ralpha: A novel target for the treatment of osteosarcoma. Adv. Exp. Med. Biol..

[13] Lokau J., Nitz R., Agthe M., Monhasery N., Aparicio-Siegmund S., Schumacher N., Wolf J., Moller-Hackbarth K., Waetzig G.H., Grotzinger J. (2016). Proteolytic cleavage governs interleukin-11 trans-signaling. Cell Rep..

[14] Xu D.H., Zhu Z., Wakefield M.R., Xiao H., Bai Q., Fang Y. (2016). The role of IL-11 in immunity and cancer. Cancer Lett..

[15] Cardo-Vila M., Marchio S., Sato M., Staquicini F.I., Smith T.L., Bronk J.K., Yin G.S., Zurita A.J., Sun M.H., Behrens C. (2016). Interleukin-11 receptor is a candidate target for ligand-directed therapy in lung cancer analysis of clinical samples and BMTP-11 preclinical activity. Am. J. Pathol..

[16] Curtis D.J., Hilton D.J., Roberts B., Murray L., Nicola N., Begley C.G. (1997). Recombinant soluble interleukin-11 (IL-11) receptor alpha-chain can act as an IL-11 antagonist. Blood.

[17] Du X.X., Williams D.A. (1997). Interleukin-11: Review of molecular, cell biology, and clinical use. Blood.

[18] Guk K.D., Kuprash D.V. (2011). Interleukin-11, an IL-6 like cytokine. Mol. Biol..

[19] Permyakov E.A., Uversky V.N., Permyakov S.E. (2016). Interleukin-11: A multifunctional cytokine with intrinsically disordered regions. Cell Biochem. Biophys..

[20] Putoczki T.L., Dobson R.C., Griffin M.D. (2014). The structure of human interleukin-11 reveals receptor-binding site features and structural differences from interleukin-6. Acta Crystallogr. D Biol. Crystallogr..

[21] Van Snick J. (1990). Interleukin-6: An overview. Annu. Rev. Immunol..

[22] Wang X.M., Wilkin J.M., Boisteau O., Harmegnies D., Blanc C., Vandenbussche P., Montero-Julian F.A., Jacques Y., Content J. (2002). Engineering and use of p-32-labeled human recombinant interleukin-11 for receptor binding studies. Eur. J. Biochem..

[23] Leon A., Wang X.M., Champion-Arnaud P., Sobczyk A., Pain B., Content J., Jacques Y., Valarche I. (2005). Expression of a bioactive recombinant human interleukin-11 in chicken HD11 cell line. Cytokine.

[24] Zhu J.K., Xu Z.X., Huang W.D., Ming T.S., Xie W., Xu Y., Zhang X.G. (2001). Expression and purification of recombinant human interleukin-11 in Pichia pastoris. Zhongguo Yi Xue Ke Xue Yuan Xue Bao.

[25] Tang J., Xu X., Nie X., Mao Q., Gao J. (2012). Prokaryotic expression, purification, and identification of recombinant human IL-11 protein. Sheng Wu Yi Xue Gong Cheng Xue Za Zhi.

[26] Farajnia S., Hassanpour R., Lotfipour F. (2010). Cloning and expression of human IL-11 in *E. coli*. Pharm. Sci..

[27] Han W., Zhang Y., Yan Z., Shi J. (2003). Construction of a new tumour necrosis factor fusion-protein expression vector for high-level expression of heterologous genes in *Escherichia coli*. Biotechnol. Appl. Biochem..

[28] Tan H., Dan G., Gong H., Cao L. (2005). Purification and characterization of recombinant truncated human interleukin-11 expressed as fusion protein in *Escherichia coli*. Biotechnol. Lett..

[29] Zhao Y., Huang H. (2008). Preparation of rhIL-11 from fusion protein by using enterokinase. Zhongguo Shi Yan Xue Ye Xue Za Zhi.

[30] Zhang Y., Liu C.H., Liu Y.L., Tien P. (2000). cDNA cloning, fusion expression in *Escherichia coli* and activity assay of hIL-11. Sheng Wu Gong Cheng Xue Bao.

[31] Morris J.C., Neben S., Bennett F., Finnerty H., Long A., Beier D.R., Kovacic S., McCoy J.M., DiBlasio-Smith E., La Vallie E.R. (1996). Molecular cloning and characterization of murine interleukin-11. Exp. Hematol..

[32] Kazakov A.S., Sokolov A.S., Rastrygina V.A., Solovyev V.V., Ismailov R.G., Mikhailov R.V., Ulitin A.B., Yakovenko A.R., Mirzabekov T.A., Permyakov E.A. (2015). High-affinity interaction between interleukin-11 and S100P protein. Biochem. Biophys. Res. Commun..

[33] Catanzariti A.M., Soboleva T.A., Jans D.A., Board P.G., Baker R.T. (2004). An efficient system for high-level expression and easy purification of authentic recombinant proteins. Protein Sci..

[34] Baker R.T., Catanzariti A.M., Karunasekara Y., Soboleva T.A., Sharwood R., Whitney S., Board P.G. (2005). Using deubiquitylating enzymes as research tools. Method Enzymol..

[35] Catic A., Misaghi S., Korbel G.A., Ploegh H.L. (2007). Elad, a deubiquitinating protease expressed by *E. coli*. PLoS ONE.

[36] Hilton D.J., Hilton A.A., Raicevic A., Rakar S., Harrison-Smith M., Gough N.M., Begley C.G., Metcalf D., Nicola N.A., Willson T.A. (1994). Cloning of a murine IL-11 receptor alpha-chain; requirement for gp130 for high affinity binding and signal transduction. EMBO J..

[37] Harmegnies D., Wang X.M., Vandenbussche P., Leon A., Vusio P., Grotzinger J., Jacques Y., Goormaghtigh E., Devreese B., Content J. (2003). Characterization of a potent human interleukin-11 agonist. Biochem. J..

[38] Ren C.L., Chen Y., Han C.X., Fu D.Y., Chen H. (2014). Plasma interleukin-11 (IL-11) levels have diagnostic and prognostic roles in patients with pancreatic cancer. Tumor Biol..

[39] Czupryn M., Bennett F., Dube J., Grant K., Scoble H., Sookdeo H., McCoy J.M. (1995). Alanine-scanning mutagenesis of human interleukin-11: Identification of regions important for biological activity. Ann. N. Y. Acad. Sci..

[40] Dunker A.K., Babu M., Barbar E., Blackledge M., Bondos S.E., Dosztányi Z., Dyson H.J., Forman-Kay J., Fuxreiter M., Gsponer J. (2013). What’s in a name? Why these proteins are intrinsically disordered. Intrinsically Disord. Proteins.

[41] Dunker A.K., Obradovic Z., Romero P., Garner E.C., Brown C.J. (2000). Intrinsic protein disorder in complete genomes. Genome Inform. Ser. Workshop Genome Inform..

[42] Ward J.J., Sodhi J.S., McGuffin L.J., Buxton B.F., Jones D.T. (2004). Prediction and functional analysis of native disorder in proteins from the three kingdoms of life. J. Mol. Biol..

[43] Tokuriki N., Oldfield C.J., Uversky V.N., Berezovsky I.N., Tawfik D.S. (2009). Do viral proteins possess unique biophysical features?. Trends Biochem. Sci..

[44] Xue B., Williams R.W., Oldfield C.J., Dunker A.K., Uversky V.N. (2010). Archaic chaos: Intrinsically disordered proteins in Archaea. BMC Syst. Biol..

[45] Uversky V.N. (2010). The mysterious unfoldome: Structureless, underappreciated, yet vital part of any given proteome. J. Biomed. Biotechnol..

[46] Xue B., Dunker A.K., Uversky V.N. (2012). Orderly order in protein intrinsic disorder distribution: Disorder in 3500 proteomes from viruses and the three domains of life. J. Biomol. Struct. Dyn..

[47] Daughdrill G.W., Pielak G.J., Uversky V.N., Cortese M.S., Dunker A.K., Buchner J., Kiefhaber T. (2005). Natively disordered proteins. Handbook of Protein Folding.

[48] Dunker A.K., Brown C.J., Obradovic Z. (2002). Identification and functions of usefully disordered proteins. Adv. Protein Chem..

[49] Dunker A.K., Brown C.J., Lawson J.D., Iakoucheva L.M., Obradovic Z. (2002). Intrinsic disorder and protein function. Biochemistry.

[50] Dunker A.K., Cortese M.S., Romero P., Iakoucheva L.M., Uversky V.N. (2005). Flexible nets. The roles of intrinsic disorder in protein interaction networks. FEBS J..

[51] Dunker A.K., Garner E., Guilliot S., Romero P., Albrecht K., Hart J., Obradovic Z., Kissinger C., Villafranca J.E. (1998). Protein disorder and the evolution of molecular recognition: Theory, predictions and observations. Pac. Symp. Biocomput..

[52] Dunker A.K., Lawson J.D., Brown C.J., Williams R.M., Romero P., Oh J.S., Oldfield C.J., Campen A.M., Ratliff C.M., Hipps K.W. (2001). Intrinsically disordered protein. J. Mol. Graph. Model..

[53] Dyson H.J., Wright P.E. (2005). Intrinsically unstructured proteins and their functions. Nat. Rev. Mol. Cell Biol..

[54] Wright P.E., Dyson H.J. (1999). Intrinsically unstructured proteins: Re-assessing the protein structure-function paradigm. J. Mol. Biol..

[55] Tompa P. (2002). Intrinsically unstructured proteins. Trends Biochem. Sci..

[56] Tompa P. (2005). The interplay between structure and function in intrinsically unstructured proteins. FEBS Lett..

[57] Tompa P., Csermely P. (2004). The role of structural disorder in the function of RNA and protein chaperones. FASEB J..

[58] Tompa P., Szasz C., Buday L. (2005). Structural disorder throws new light on moonlighting. Trends Biochem. Sci..

[59] Uversky V.N. (2002). Natively unfolded proteins: A point where biology waits for physics. Protein Sci..

[60] Uversky V.N. (2002). What does it mean to be natively unfolded?. Eur. J. Biochem..

[61] Uversky V.N., Gillespie J.R., Fink A.L. (2000). Why are “natively unfolded″ proteins unstructured under physiologic conditions?. Proteins.

[62] Uversky V.N. (2003). Protein folding revisited. A polypeptide chain at the folding-misfolding-nonfolding cross-roads: Which way to go?. Cell. Mol. Life Sci..

[63] Xie H., Vucetic S., Iakoucheva L.M., Oldfield C.J., Dunker A.K., Uversky V.N., Obradovic Z. (2007). Functional anthology of intrinsic disorder. 1. Biological processes and functions of proteins with long disordered regions. J. Proteome Res..

[64] Vucetic S., Xie H., Iakoucheva L.M., Oldfield C.J., Dunker A.K., Obradovic Z., Uversky V.N. (2007). Functional anthology of intrinsic disorder. 2. Cellular components, domains, technical terms, developmental processes, and coding sequence diversities correlated with long disordered regions. J. Proteome Res..

[65] Xie H., Vucetic S., Iakoucheva L.M., Oldfield C.J., Dunker A.K., Obradovic Z., Uversky V.N. (2007). Functional anthology of intrinsic disorder. 3. Ligands, post-translational modifications, and diseases associated with intrinsically disordered proteins. J. Proteome Res..

[66] Uversky V.N., Oldfield C.J., Dunker A.K. (2005). Showing your ID: Intrinsic disorder as an ID for recognition, regulation and cell signaling. J. Mol. Recognit..

[67] Cheng Y., Oldfield C.J., Meng J., Romero P., Uversky V.N., Dunker A.K. (2007). Mining alpha-helix-forming molecular recognition features with cross species sequence alignments. Biochemistry.

[68] Dunker A.K., Silman I., Uversky V.N., Sussman J.L. (2008). Function and structure of inherently disordered proteins. Curr. Opin. Struct. Biol..

[69] Dunker A.K., Uversky V.N. (2008). Signal transduction via unstructured protein conduits. Nat. Chem. Biol..

[70] Mohan A., Oldfield C.J., Radivojac P., Vacic V., Cortese M.S., Dunker A.K., Uversky V.N. (2006). Analysis of molecular recognition features (MoRFs). J. Mol. Biol..

[71] Oldfield C.J., Cheng Y., Cortese M.S., Romero P., Uversky V.N., Dunker A.K. (2005). Coupled folding and binding with alpha-helix-forming molecular recognition elements. Biochemistry.

[72] Laemmli U.K. (1970). Cleavage of structural proteins during the assembly of the head of bacteriophage T4. Nature.

[73] Pace C.N., Vajdos F., Fee L., Grimsley G., Gray T. (1995). How to measure and predict the molar absorption coefficient of a protein. Protein Sci..

[74] Romero P., Obradovic Z., Li X., Garner E.C., Brown C.J., Dunker A.K. (2001). Sequence complexity of disordered protein. Proteins.

