# Supplementary material for: Expression, Purification, and Characterization of Interleukin-11 Orthologues
Source: Molecules. 2016 Nov 29;21(12):1632. doi: 10.3390/molecules21121632 (PMC6274577; doi:10.3390/molecules21121632)
Supplement: Supplementary file 1 [file molecules-21-01632-s001.pdf]

Supplementary Materials: Expression, Purification, and Characterization of Interleukin-11 Orthologues

Andrei S. Sokolov, Alexei S. Kazakov, Valery V. Solovyev, Ramis G. Ismailov, Vladimir N. Uversky, Yulia S. Lapteva, Roman V. Mikhailov, Ekaterina V. Pavlova, Iana O. Terletskaya, Ludmila V. Ermolina, Sergei E. Permyakov and Eugene A. Permyakov

Table S1. The nucleotide sequences encoding mature IL-11 from *Macaca fascicularis* (№1) and *Mus musculus* (№2) lacking Pro1 residue (Swiss-Prot entries P20808 and P47873, respectively). The corresponding protein sequences and their alignment with human IL-11 are shown in Figure S1.

№	Nucleotide sequence
1	ggtccgccgccaggtagccctcgtgcaagcccggatccgcgtgctgaactggactccactgtgctgctgacgcgtagcctcctggaagatactcgtc<br>agctgactatccaactgaaagacaaattcccggctgatggtgaccacaacctggattctctgccgactctggctatgagcgcgggcgcgctgggtgc<br>gctgcaactgccatctgttctgacccgtctgcgtgcagacctgctgagctacctgcgccacgtccagtggctgcgtcgtgcgatgggcagctctctga<br>aaaccctggagccggaactgggtaccctgcagacccgtctggaccgcctgctgcgccgtctgcagctgttgatgtctcgcctggccctgcctcagctg<br>ccgcctgatccgccagctccaccgctggccccgccgagctccacctggggtggtattcgtgcagcacacgcaatcctgggcggtctgcacctgaccc<br>tggactgggcggtacgtggtctgcttctgctcaaaactcgcctgtaa
2	ggtccaccggcaggctctcctcgtgtctcctccgacccccgcgcagatctggattctgcggtgttactgacccgtagtctgctggctgatacccgccag<br>ctggctgcccagatgcgtgataaatttcctgccgacggcgatcattcactggatagcctgccgactctcgccatgtcagcaggcaccctcggctctctg<br>cagttaccgggtgtgctgactcgcctgcgtgttgatctgatgagttatttacgccatgtacagtggcttcgccgtgcaggtggtccctccctgaaaacct<br>tagagccggagctgggtgccctgcaggcacgtctggaacgtctcctgcgtcgtctgcaactcttgatgtcgcgcctggccctgccccaggcggctcc<br>ggatcagccggtaatcccgctcggtccgcctgcatcagcatgggggagtattcgcgctgctcatgcgattctgggcggactgcaccttacccttgatt<br>gggccgtgcgcgggctgctgcttctgaaaactcggctttaa

Table S2. Nucleotide sequences of the primers used for cloning of macaque and mouse IL-11 genes (Table S1) into pHUE vector between the *Sac*II and *Not*I restriction sites (underlined).

№	Primer name	5' to 3' end sequence
1	IL-11 macaque *Sac*II	atct<u>ccgcgg</u>tggtggtccgccgccagg
2	IL-11 mouse *Sac*II	atct<u>ccgcgg</u>tggtggtccaccggcaggc
3	IL-11 macaque stop *Not*I	tatt<u>gcggccgc</u>attacaggcgagttttgagcagaag
4	IL-11 mouse stop *Not*I	tatt<u>gcggccgc</u>attaaagccgagttttcagaagc

Table S3. ESI-MS data for macaque and murine rIL-11 samples, estimated from the data shown in Figure 4.

Protein	Molecular mass of the component (relative content)	Description of the component
Macaque rIL-11	19,292 Da (49%)	corresponds to the predicted mass
	19,313 Da (21%)	one sodium atom is bound
	19,390 Da (20%)	unassigned component
	19,930 (10%)	unassigned component
Murine rIL-11	19,057 Da (48%)	corresponds to the predicted mass
	19,080 Da (25%)	one sodium atom is bound
	19,103 Da (14%)	two sodium atoms are bound
	19,695 Da (12%)	unassigned component

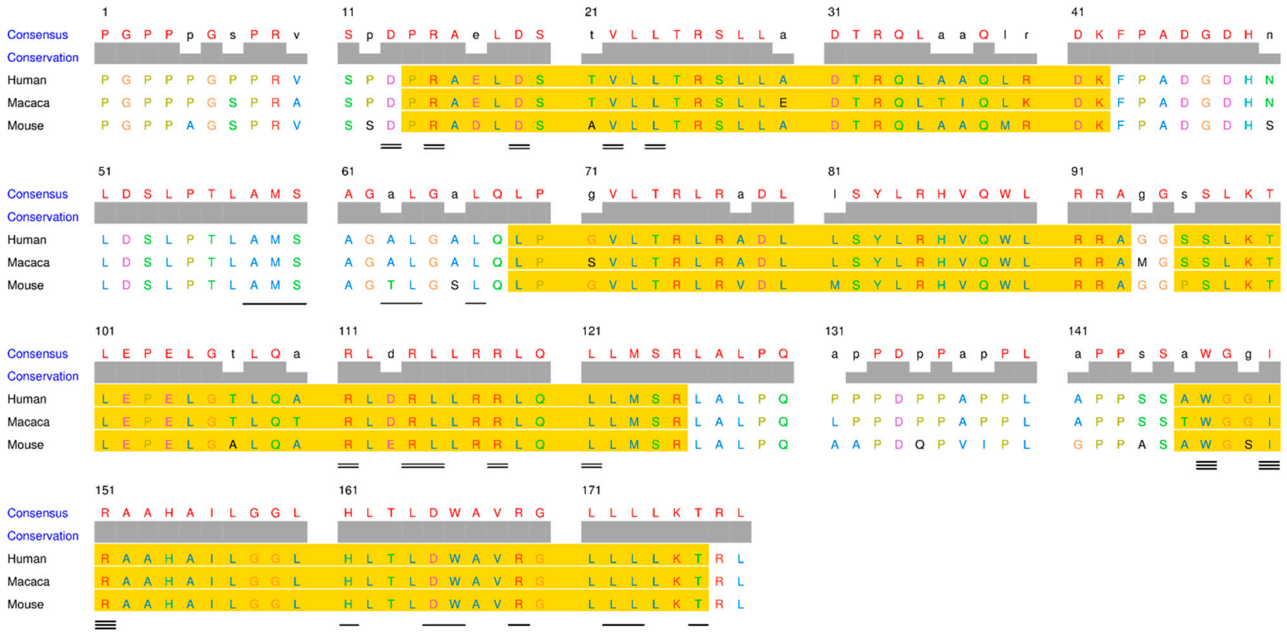

Figure S1. Conservation analysis of mature IL-11 orthologs from human, macaque and mouse (Swiss-Prot entries P20809, P20808 and P47873, respectively). The protein sequences were aligned using online Clustal Omega service (http://www.ebi.ac.uk/Tools/msa/clustalo/). The alignment and sequence conservation are visualized using UCSF Chimera v.1.10.2 software. α-Helical regions are marked as orange filled rectangles. The sites proposed for human IL-11 interaction with IL-11Rα (site I, single underline) and gp130 (site II, double underline; site III, triple underline) receptors [1] are indicated (see Figure 1).

>sp|P20809|IL11_HUMAN Interleukin-11 OS=Homo sapiens GN=IL11 PE=1 SV=1, residues 22-199
PGPPPGPPRVSPDPRAELDSTVLLTRSLLADTRQLAAQLRDKFPADGDHNLDSLPTLAMSAGALGA
LQLPGVLTRLRADLLSYLRHVQWLRRAGGSSLKTLEPELGTLQARLDRLLRRLQLLMSRLALPQPP
PDPPAPPLAPPSSAWGGIRAAHAILGGLHLTLDWAVRGLLLLKTRL

>sp|P20808|IL11_MACFA Interleukin-11 OS=Macaca fascicularis GN=IL11 PE=2 SV=1, residues 22-199
PGPPPGSPRASPDPRAELDSTVLLTRSLLEDTRQLTIQLKDKFPADGDHNLDSLPTLAMSAGALGA
LQLPSVLTRLRADLLSYLRHVQWLRRAMGSSLKTLEPELGTLQTRLDRLLRRLQLLMSRLALPQLP
PDPPAPPLAPPSSTWGGIRAAHAILGGLHLTLDWAVRGLLLLKTRL

>sp|P47873|IL11_MOUSE Interleukin-11 OS=Mus musculus GN=Il11 PE=1 SV=1, residues 22-199
PGPPAGSPRVSSDPRADLDSAVLLTRSLLADTRQLAAQMRDKFPADGDHSLDSLPTLAMSAGTLGS
LQLPGVLTRLRVDLMSYLRHVQWLRRAGGPSLKTLEPELGALQARLERLLRRLQLLMSRLALPQAA
PDQPVIPLGPPASAWGSIRAAHAILGGLHLTLDWAVRGLLLLKTRL

>sp|P05231|IL6_HUMAN Interleukin-6 OS=Homo sapiens GN=IL6 PE=1 SV=1, residues 26-212
FPAPVPPGEDSKDVAAPHRQPLTSSERIDKQIRYILDGISALRKETCNKSNMCESSKEALAENNLN
LPKMAEKDGCFQSGFNEETCLVKIITGLLEFEVYLEYLQNRFESSEEQARAVQMSTKVLIQFLQKK
AKNLDAITTPDPTTNASLLTKLQAQNQWLQDMTTHLILRSFKEFLQSSLRALRQM

>sp|P79341|IL6_MACFA Interleukin-6 OS=Macaca fascicularis GN=IL6 PE=2 SV=1, residues 26-212
FPAPVLPGEDSKDVAAPHSQPLTSSERIDKHIRYILDGISALRKETCNRSNMCESSKEALAENNLN
LPKMAEKDGCFQSGFNEDTCLVKIITGLLEFEVYLEYLQNRFESSEEQARAVQMSTKVLIQFLQKK
AKNLDAITTPEPTTNASLLTKLQAQNQWLQDMTTHLILRSFKEFLQSSLRALRQM

>sp|P08505|IL6_MOUSE Interleukin-6 OS=Mus musculus GN=Il6 PE=1 SV=1
FPTSQVRRGDFTEDTTPNRPVYTTSQVGGLITHVLWEIVEMRKELCNGNSDCMNNDDALAENNLKL
PEIQRNDGCYQTGYNQEICLLKISSGLLEYHSYLEYMKNNLKDNKKDKARVLQRDTETLIHIFNQE
VKDLHKIVLPTPISNALLTDKLESQKEWLRTKTIQFILKSLEEFLKVTLRSTRQT

Figure S2. Amino acid sequences of mature forms of IL-11 and IL-6 from *Homo sapiens*, *Macaca fascicularis*, and *Mus musculus*

Reference

1. Putoczki, T.L.; Dobson, R.C.; Griffin, M.D. The structure of human interleukin-11 reveals receptor-binding site features and structural differences from interleukin-6. *Acta Crystallogr. D Biol. Crystallogr.* **2014**, *70*, 2277–2285.
